# SLEEP DURATION AND COGNITION IN THE LONG LIFE FAMILY STUDY

**DOI:** 10.1093/geroni/igae098.0031

**Published:** 2024-12-31

**Authors:** Emma Gordon, Yian Gu, Stephanie Cosentino, Stacy Anderson, Svetlana Ukraintseva

**Affiliations:** Albert Einstein College of Medicine, Bronx, New York, United States; Columbia University, New York, New York, United States; Columbia University Irving Medical Center, New York, New York, United States; Boston University, Boston, Massachusetts, United States; Duke University, Durham, North Carolina, United States

## Abstract

Numerous studies demonstrate associations between sleep and cognition. This study includes a subset of 2344 participants from the Long Life Family Study, a cohort with slower overall rates of cognitive decline, and seeks to examine how sleep and cognition are related in this population. Our neuropsychological battery measured episodic, semantic, and working memory at baseline (2006-2009), and in a subset of 1633 participants at the follow-up visits 6.9 (SD=2.7) years later. Sleep habits were assessed once, using the Health Habits (HH) Questionnaire, between the two cognitive measures. Associations between sleep duration and memory were examined using generalized estimating equations models, accounting for relationship with long-lived families. Compared to those with 6.5-8.5 hours of sleep, those with > 8.5 hours of sleep had lower episodic memory scores at baseline (b=-0.705, p=0.021), but not at follow-up (b=-0.627, p=0.149), after controlling for age, sex, field center, APOE, and generation. Sleep duration was not associated with semantic or working memory. Those who had > 8.5 hours of sleep had faster decline in all memory scores, although not significantly. There was no difference in memory scores comparing < 6.5 hours of sleep to 6.5-8.5 hours of sleep. Lower memory scores at baseline predicted longer sleep time subsequently, while sleep time did not predict memory performance on follow-up. Overall, the current study did not support sleep duration as a significant contributor to memory decline, contradicting some earlier findings in the literature, and prompting further investigation into the connection between sleep and cognition, especially within this population.

